# Epidemiology and Treatment of Surgical Infection after Ankle Arthroscopy: A Systematic Review

**DOI:** 10.3390/jcm13040983

**Published:** 2024-02-08

**Authors:** Andrea De Fazio, Maria Beatrice Bocchi, Guglielmo Miele, Pasquale Ruberto, Fabrizio Forconi, Antonio Ziranu, Giulio Maccauro, Raffaele Vitiello

**Affiliations:** 1 Department of Orthopaedics and Traumatology, Fondazione Policlinico Universitario A. Gemelli IRCSS, 00168 Roma, Italy; andrea.defazio01@icatt.it (A.D.F.); mariabeatrice.bocchi01@icatt.it (M.B.B.); pasquale.ruberto01@icatt.it (P.R.); antonio.ziranu@policlinicogemelli.it (A.Z.); giulio.maccauro@policlinicogemelli.it (G.M.); raffaele.vitiello@guest.policlinicogemelli.it (R.V.); 2 Department of Orthopaedics and Traumatology, Università Cattolica Del Sacro Cuore, 00168 Roma, Italy; 3 Clinic Villa Stuart, 00135 Rome, Italy; fabrizioforconi@gmail.com

**Keywords:** infection, arthroscopy, ankle, ankle arthroscopy

## Abstract

**Background**: Ankle arthroscopy is indicated for both diagnosis and treatment of a large spectrum of common ankle disorders. It has certain advantages over the open procedure; however, it is important to recognize that there are some complications associated with it. Infections after this procedure are quite uncommon, with an overall estimated incidence of 2%. Given the low incidence of infections after ankle arthroscopy, not a great deal of literature on the topic has been published. The present review aims to provide an overview of the incidence, diagnosis, and treatment of infections after ankle arthroscopy. **Methods**: A systematic review of the literature indexed in the PubMed, MEDLINE, and Cochrane Library databases using search term “ankle arthroscopy infections” was performed in November 2023. No restrictions were applied concerning the date of publication. The Preferred reporting items for systematic reviews and meta-analyses (PRISMA) were followed. Among all surgical operations for the treatment of ankle and foot pathologies, we included articles with a described superficial or deep infection after ankle arthroscopy. **Results:** The search resulted in 201 studies. Only 21 studies met our inclusion criteria, and they were included in this systematic review. We evaluated 1706 patients who underwent 1720 arthroscopic tibiotalar procedures at an average age of 42 years old. Out of the 1720 procedures, 41 (2%) were complicated by infection. We divided infectious complications into superficial (68%; 28/41) and deep (32%; 13/41) infections. The most common pathogen isolated was Staphylococcus aureus. Arthroscopic arthrodesis was found to be the most affected by deep infections. **Conclusions**: Infection after ankle arthroscopy is an uncommon complication. Superficial infections were successfully treated with antibiotics, while surgical debridement, arthroscopic drainage, and intravenous antibiotics were necessary in cases of deep infections. Considering the amount of information on pathogens associated with knee and shoulder infections, there is still a lack of literature on pathogens associated with ankle infections, which makes their management difficulty.

## 1. Introduction

Takagi was the first surgeon to perform an arthroscopic examination of a joint using a cystoscope 56 to directly visualize a cadaver knee joint. In 1939, Takagi published the results of his work, describing a case of ankle arthroscopy of a flail ankle [1]. In the early 1930s, Burman conducted an experimental cadaver study where he performed arthroscopy in joints other than the knee. In this study, Burman stated that the ankle joint was not suited for arthroscopy, since the articular space was too narrow and the large instruments of the day made this procedure impossible. Burman also described how he could not accomplish any separation in the ankle joint despite traction [2,3]. While Burman was working in New York, one of his visitors was the Japanese surgeon Watanabe, who, in 1970, developed the fiberoptic arthroscope, which allowed him to perform 28 ankle arthroscopies described in a study he published in 1972. Watanabe described the standard anterolateral, anteromedial, and posterior arthroscopic portals of the ankle for the first time [4]. Nowadays, foot and ankle surgeons are increasingly interested in arthroscopy. The technological advancements in this field have allowed surgeons to treat an increasing number of ankle pathologies with an arthroscopic approach. Ankle arthroscopy is indicated for both the diagnosis and treatment of a large spectrum of common ankle disorders, such as osteochondral lesions, soft tissue and bony ankle impingement, ankle instability, loose body removal, chondral or syndesmosis injury, arthritis, chronic synovitis, ankle arthrodesis, acute trauma, and sequelae [5]. Ankle arthroscopy is usually performed using the standard anteromedial and anterolateral portals [6]. About 20 years ago, two posterior portals were also proposed to treat posterior impingement [7]. During ankle arthroscopy procedures, the joint distraction technique is important to successfully overcome the narrowness of the ankle joint, allowing the surgeon to correctly visualize the ankle joint space. Recently, there has been a shift from invasive to noninvasive distraction due to its excellent results [8]. Compared to open surgery, ankle arthroscopy treatment has a lot of advantages. First of all, it requires small, minimally invasive incisions to guarantee an adequate exposure. In addition, following ankle arthroscopy, the patient has low postoperative morbidity, less blood loss, a shorter hospital stay, faster rehabilitation and mobilization, and a low complication rate, and the procedure can be performed even in patients with both poor skin and wound-healing potential, which would be a contraindication to the open technique. Again, during ankle arthroscopy, the surgeon can directly visualize the intra-articular structure without causing extensive soft tissue damage. In addition, the surgeon can test the joint in case of laxity and evaluate the ligamentous structures of the ankle. [9]. However, this procedure is challenging, with a long learning curve, and it is not without complications, considering the small volume of space in which it must be performed [2]. Several studies have investigated the incidence of complications following ankle arthroscopy [10,11]. In the 1990s, Small et al. reported a complication rate in knee and other joint arthroscopic procedures of 0.5–0.6%, which is low compared with all other data in the literature [12,13]. In a study published in 2001, Ferkel et al. reported a complication rate in ankle arthroscopy of around 9% [9]. This value was significantly lower than what was described by the scientific literature, as previous studies reported complication rates in ankle arthroscopy ranging from 15% to 17% [3,14]. According to the latest meta-analysis, the total incidence of complications is around 4.09%. Ankle arthroscopy-related complications can be categorized as systemic, preoperative, and procedure-related. Systemic complications are related to anesthesia, patient illness, and surgical stress; they include cardiopulmonary events, atelectasis, pulmonary embolus, and myocardial infarction. Preoperative complications include incorrect diagnosis with consequent incorrect preoperative planning. Procedure-related complications are strictly dependent on the surgery performed [9]. The most frequent of such complications is neurological damage, representing 55.4% of all complications [10]. Injuries involving the superficial peroneal nerve are most common, followed by injuries to the deep peroneal nerve, while injuries to the sural and saphenous nerves are less common [10]. Other complications have been reported, such as infection, extrinsic articular cartilage damage, compartment ischemia, tendon and ligament injuries, postoperative swelling, deep vein thrombosis, and complex regional pain syndrome [8,9]. Some authors believe that there are several factors that increase the complication rate following ankle arthroscopy, such as the use of a tourniquet, as well as the use of a high-volume fluid pump; postoperative corticosteroid injection; the perioperative injection of anesthetics; skin closure technique (suture, tape, or clip); prolonged operative time; changing the patient’s setup up from the prone to the supine position or vice-versa; and the surgeon’s expertise [8,15,16,17,18,19]. The incidence of infections after ankle arthroscopy has been reported to be between 0.13 and 2% [10,12,16,17]. Although it is reasonable to assume that comorbidities such as diabetes, peripheral neuropathy, peripheral vascular disease, tobacco use, and high values of the body mass index can increase the risk of infections, it seems there are no statistically significant relations between these conditions and ankle arthroscopy complication rates [20,21]. Ankle arthroscopy-related infections can occur either as superficial or as deep to the point of involving the entire joint. Superficial infections can be treated with antibiotic therapy. Deep infections, on the other hand, require either further arthroscopy with lavage or a second open surgery to debride the tissues after any failure of medical treatment. [8,9,22]. Ankle arthroscopy seems to have a higher incidence of complication than arthroscopy performed in other joints, such as the knee [3]; this may be due to the close association between many anatomical structures in a narrow space, both anterior and posterior [14]. Also, the thin soft tissue coverage of the ankle can predispose this area to both superficial and deep infections [23]. To date, there have been very few reports dealing with the incidence of infections following ankle arthroscopy. Also, most articles on this matter do not provide thorough details about the antibiotic used or the surgical procedure performed to treat the infection. The present review aims to provide an overview the incidence, diagnosis, and treatment of infections after ankle arthroscopy.

## 2. Materials and Methods

### 2.1. Search Strategy

A systematic review of the literature indexed in the PubMed, MEDLINE, and Cochrane Library databases using the search term “ankle arthroscopy infections” was performed in November 2023. No filters were applied to the search strategy to minimize the number of missed studies. The bibliographies of the selected studies were accurately searched by hand to identify further studies not found during the electronic search. No restrictions were applied concerning the date of publication. The journal’s title, the author’s name, or supporting institutions were not masked at any stage. The preferred reporting items for systematic reviews and meta-analyses (PRISMA) were followed. This study was not registered; therefore, there is no registration number. This article adheres to the latest preferred reporting items for systematic reviews and meta-analyses statement [24]; to be considered for this review, the articles needed to present some inclusion criteria: at least a section of the population under study had to be affected by accidental infection following arthroscopic tibiotalar surgery only. Furthermore, only full-text articles in the English language were considered. Review articles, cadaveric and animal studies, and non-cohort were excluded.

### 2.2. Study Selection

Two independent reviewers (G.M. and P.R.) performed the literature search and reviewed the results. The titles and abstracts were reviewed for all search results, and potentially eligible studies received a full-text review. All reviewers’ differences were discussed, and the senior author (R.V.) was consulted if disagreement remained.

### 2.3. Data Extraction/Analysis

All the selected studies were retrospectively analyzed by an author (A.D.F.), who then extracted and entered the data in an Excel worksheet (Microsoft Corporation, Redmond, WA, USA. Microsoft Excel [Internet]. 2018. Available from: https://office.microsoft.com/excel). The collected data included the primary author, year of publication, article type, number of patients and procedures performed, age, initial diagnosis, arthroscopic procedure performed, number and type of infections diagnosed, possible infectious agent isolated, and, finally, treatment undertaken. Lastly, the data sheet was reviewed by two authors (M.B.B. and R.V.), who agreed on the extracted data. The methodological quality of the studies was assessed using the modified Coleman Methodology Score (mCMS) [25]. Two independent investigators evaluated each article (A.D.F. and M.B.B.); in cases with more than a five-point difference between their ratings, the discrepancy was solved by consensus with a third author (R.V.). The mCMS ranges from 0 to 100 points, representing a well-designed study with no bias or confounding factors. In further detail, the final score is categorized as excellent (85–100 points), good (70–84 points), fair (50–69 points), or poor (<50 points).

## 3. Results

### 3.1. Search and Literature Selection

**Figure 1:**
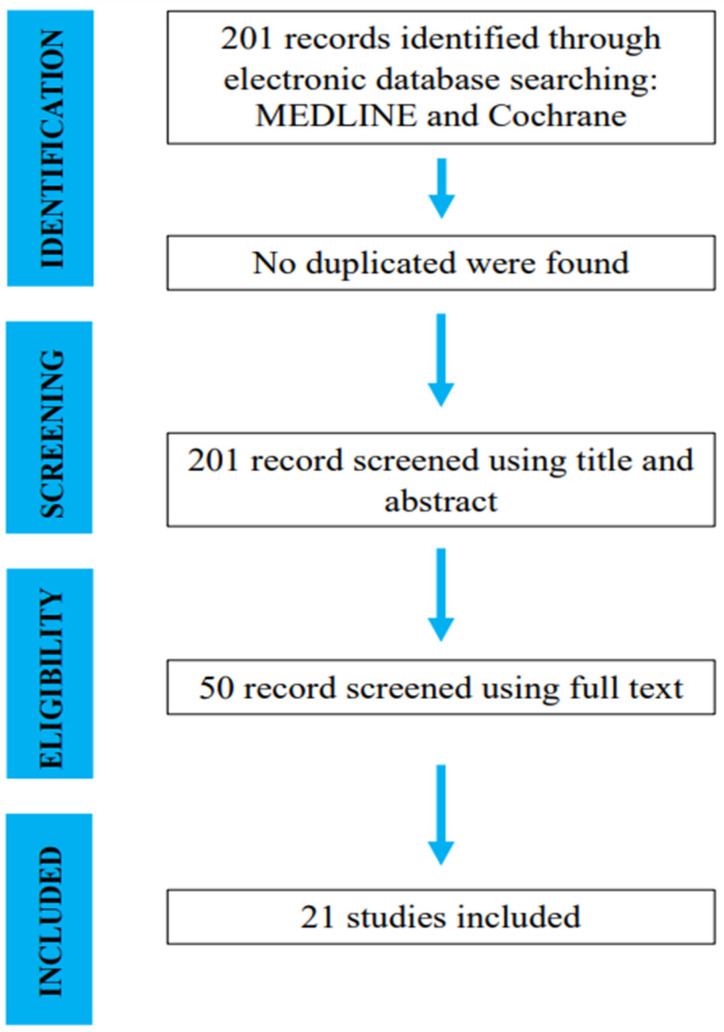
Search and literature selection.

The initial literature search resulted in 201 studies. Once duplicates were removed and the articles were screened for inclusion and exclusion criteria, 50 studies remained, and full texts were assessed for eligibility (Figure 1).

### 3.2. Study Characteristics

Twenty-one articles were included in this systematic review [3,8,11,14,19,23,26,27,28,29,30,31,32,33,34,35,36,37,38,39,40]. Of the 21 studies, 13 (62%) were retrospective, and 8 (38%) were prospective studies. Quality scoring through the modified Coleman Methodology Score was then assessed, and the mean score of the studies was 53 points (42–65 points), representing a fair study design.

We evaluated 1706 patients who had undergone 1720 arthroscopic tibiotalar procedures at an average age of 42 (Table 1).

### 3.3. The Initial Diagnosis

**Figure 2:**
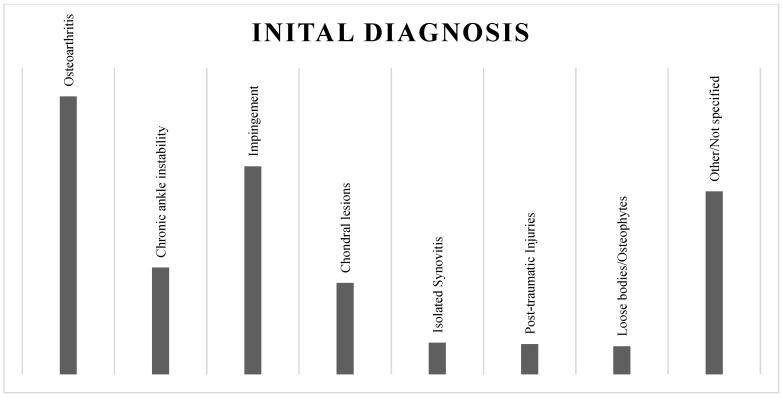
Initial diagnosis.

Most patients were indicated to perform ankle arthroscopy for osteoarthritis (544). The most encountered diagnoses were chronic ankle instability (209), anterior (377) and posterior (30) impingement, chondral lesions (179), isolated synovitis (62), post-traumatic injuries (59), loose bodies and osteophytes (55), and other or not specified tibiotalar pathologies (358) (Figure 2).

### 3.4. Ankle Arthroscopy

Among all the tibiotalar arthroscopies performed, the anterior approach was used in most cases (67%; 1157/1720), either with anteromedial, anterolateral, or anterocentral portals. The posterior approach was chosen only for a few procedures (3%; 49/1720). A combined approach was used in 30% of ankle arthroscopies (514/1720).

**Figure 3:**
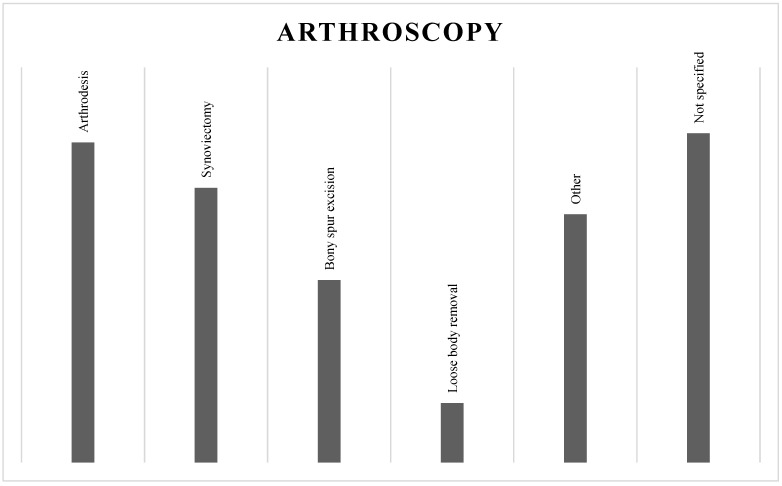
Ankle arthroscopy performed.

Arthroscopic arthrodesis (486) was found to be the most performed procedure, followed by synovectomy (417), bony spur excision (277), loose body removal (90), and others (377). In some cases, the procedure performed was not explicit (500) (Figure 3).

### 3.5. Infection Rate

Out of the 1720 procedures, 41 (2%) were complicated by infection. We divided infectious complications into superficial (68%; 28/41) and deep (32%; 13/41) infections. The isolated pathogen was made explicit in four cases (10%; 4/41); in all cases, it was Staphylococcus Aureus.

All instances of superficial infections were successfully treated with medical therapy: 11 (39%) patients with oral antibiotic therapy and local care, 2 (7%) with local antibiotic therapy and care, 2 (7%) patients only with local care, and 13 (47%) cases in which the undertaken medical treatment was not specified in detail (Table 2). On the other hand, regarding cases of deep infection, two (15%) patients were treated with medical therapy and specifically intravenous antibiotic therapy, four (31%) patients were surgically treated, and six (46%) patients received antibiotic intravenous therapy and underwent surgery. Lastly, in one (8%) case, the undertaken treatment was not specified. Among all ten patients diagnosed with deep infection who underwent surgical debridement and irrigation, four (40%) patients were treated with a new arthroscopy, two (20%) patients underwent open surgery, and, finally, in four (40%) cases, the type of surgery performed was not specified (Table 3).

## 4. Discussion

In 1931, Burman stated that the ankle was inappropriate for arthroscopy because the joint space was too narrow [2]. In 1972, Watanabe introduced a No. 24 arthroscope for small joints, which is indispensable for treating ankle disorders in arthroscopy [8]. By that date, the indication for the use of ankle arthroscopy was increasing. Surgeons were adopting this technique for various conditions, such as osteochondral lesions, ankle impingement or instability, loose bodies, chondral or syndesmosis injury, arthritis, chronic synovitis, acute trauma, and sequelae [5]. Generally, it is performed with the patient in the supine position, using the anterolateral portal first, then the anteromedial one. A tourniquet is applied for hemostasis. At the end of the procedure, these two portals can be sutured [1,2,5,8,10].

Advantages of the arthroscopic technique over open techniques include low postoperative morbidity, less blood loss, shorter hospital stay, faster rehabilitation and mobilization, a low complication rate, and that it can be performed even in patients with both poor skin and wound-healing potential, which would be a contraindication to the open technique. Performing ankle arthrodesis arthroscopically also prevents limb-threatening complications and reduces union time [34].

Qualye J et al. compared 29 open and 50 arthroscopic ankle fusions [28]. They reported a higher union rate and a shorter time to fusion in the arthroscopic group, but they could not demonstrate a difference in the length of hospital stay.

Jerosch J et al. also reported reduced pain and swelling after conventional open arthrodesis [41].

Despite the advantages associated with this procedure, it is not free of complications. Several studies have investigated the rate of complications related to ankle arthroscopy, ranging from 0.7% to 17% [13,14,42].

Of course, the highest incidence rates are reported in older studies due to the limited experience of surgeons and the extensive use of invasive distraction devices to overcome the narrowness of the ankle joint [13].

According to the latest reviews, the rate of complications is 4.8% [10]. In addition, there has been a shift from invasive to noninvasive distraction because the second technique is considered safe and effective for ankle arthroscopy [8].

The most common complication is neurological injury due to the proximity of the nerves to the portals used during arthroscopy [10,22]. Among other complications, infections certainly play a crucial role. Infections after this procedure are rare but not exceptional. Data from the literature assess an incidence rate ranging from 0% to 1.8% in patients undergoing simple arthroscopic ankle procedures [10,13,17,22].

Thus, the infection rate is higher than that reported in the literature for knee and shoulder arthroscopy, at around 0.22 and 0.85%, respectively [15].

Generally, infections are divided into superficial and deep infections. Surgical-site infections (SSIs) are defined by the European Centre for Disease Prevention and Controls as microbial contamination of the surgical wound within 30 days of an operation or one year after surgery if an implant is placed in a patient [43]. Sometimes, they may be superficial infections, i.e., limited to the epidermidis; other times, they may be deep infections affecting the subcutaneous tissue, joints, organs, or implanted materials. In 2016, the CDC changed the time frame for their definition of deep surgical site infection (SSI) from within 1 year to within 90 days of surgery [44].

Based on the literature, patients presenting superficial or deep infections show local signs, such as redness, swelling, secretion from the wound, fever, and pain [15,45,46].

Early diagnosis is crucial, and it is performed based on clinical features; blood test; RMN\TC; a joint puncture for microbiological evaluation; and, eventually, revision arthroscopy, collecting tissue samples for further microbial investigation. Regarding treatment, the use of antibiotic oral therapy for superficial infections may be sufficient. For deep infections, intravenous therapy and, eventually, revision surgery are often necessary [28,46].

Although a growing interest in ankle arthroscopy has been observed among foot and ankle surgeons, precise guidelines about this topic have yet to be established.

In our review, most patients were indicated for ankle arthroscopy for osteoarthritis and ankle instability. Arthroscopic arthrodesis was the most performed procedure, followed by synovectomy and bony spur excision. The infection rate was 2% (41 cases out of 1720 procedures); 68% were superficial, and 32% were deep.

The superficial infections were localized to the arthroscopic portals or at the entry point of the screws used to perform the arthrodesis procedures [36]. In the literature, there are several techniques for arthroscopic portal suturing.

Thein R. et al. performed nine arthroscopies in cases of sports-related synovitis of the ankle joint. They reported just one complication, which was a superficial infection at one of the arthroscopic portals that was purposely left unsutured at the end of the operation. Since then, they have preferred to suture all arthroscopic wounds around the ankle joint [23].

Rolf C. et al. performed 112 arthroscopies [38]. They preferred to strip the portals instead and had just one deep infection.

Bai Z. et al. treated ten patients with arthroscopically assisted ankle arthrodesis and used absorbable sutures for portal suturing [30].

Regarding arthroscopic arthrodesis, infection of the screw’s entry point resulted in its removal. Winson IG. et al. reviewed 116 patients. They noted three superficial infections at the arthroscopic portals, and 22 cases required screw removal due to their lengths [35].

Cameron SE. et al. reported superficial infection in two patients [37]. Both involved a laterally placed screw through the fibula, and both required the removal of the screw for that reason.

All reported superficial infections were resolved in each case after a short course of oral or topical antibiotics (Bacitracin) [11] without additional surgical treatment or hospitalization.

Concerning the 13 reported deep infections, Martin DF. et al. ran 100 arthroscopies and reported two deep infections [3]. One was treated with intravenous antibiotics; the other needed revision surgery for irrigation and debridement. Also, in this case, the portals were not sutured.

Rasmussen S. et al. performed 105 arthroscopies for the treatment of anterior ankle impingement. They reported four deep infections that were treated successfully with arthroscopic synovectomy and intravenous antibiotics [19].

As we reported, these deep infections were treated heterogeneously at the surgeon’s discretion, as there are no guidelines yet in the literature. While for superficial infections, antibiotics were enough, for deep infections, another surgery had to be performed in 10 out of 14 cases. Most of the patients received open treatment with debridement and irrigation of the infected area [22]. Neither patient developed osteomyelitis or had a recurrence of the infection in the follow-up period.

Based on our review, open and arthroscopic techniques have never been compared in the treatment of deep infection following ankle arthroscopy. In a recent review found in the literature, Voss A. et al. stated a therapeutic approach for postoperative septic arthritis after arthroscopy [46]. If an infection is confirmed or suspected, it is recommended to perform an early arthroscopic joint irrigation and debridement, while if a severe infection is already diagnosed, an open revision procedure should be performed.

Regarding risk factors, only a few authors of publications in our review provided evidence of a correlation between the type of surgery performed or patient-related factors and the occurrence of infection.

Quayle J. et al. reported that among 92 patients undergoing arthroscopic arthrodesis, those with a low BMI had a higher infectious risk [28].

Thein et al. stated that thin soft-tissue coverage over the ankle joint can predispose this area to superficial or deep infection [23]. In addition, Wener et al. demonstrated that intra-articular corticosteroid injection raises the incidence of infection to 3.9% [18,47]

No author described the clinical manifestations of these infections and whether it was necessary to perform RMN or TC. Only six out of the twenty-one studies we included specified that prophylactic antibiotics were respected. In just one study, it was specified that antibiotics were not administered [3]. In all other cases, this information was not specified.

The isolated pathogen was been made explicit in four cases (10%); in all cases, it was Staphylococcus Aureus. This germ is also responsible for knee infection after knee arthroscopy and is the most common. It is also frequently isolated in shoulder infections. While there is a lot of information about germs related to knee or shoulder infections, the literature is still lacking for those involved in ankle infections, making it difficult to treat them [46].

Identifying risk factors associated with infection could provide the necessary information to treat patients properly and aid surgeons in selecting targeted preventive measures. Establishing guidelines for treating deep infection following ankle arthroscopy is also required. Even today, open surgery is still preferred, even though the results of arthroscopy are encouraging [19,22,37,46].

## 5. Conclusions

A growing interest in ankle arthroscopy has been noted among foot and ankle surgeons. Ankle arthroscopy is increasingly suggested for diagnosing and treating a large spectrum of common ankle disorders. Infections after ankle arthroscopy are rare but nonexceptional. The superficial infections were successfully treated with antibiotics, while surgical debridement, arthroscopic drainage, and intravenous antibiotics were necessary in cases of deep infections. No patient developed osteomyelitis or had a recurrence of the infection in the follow-up period. Considering the amount of information about germs related to knee or shoulder infections, the literature is still lacking for those involved in ankle infections, making it difficult to treat them. It is required to establish guidelines for the diagnosis and treatment of deep infection following ankle arthroscopy.

Limitation: The present study has some limitations, mainly because of the type of articles that were the subject of this systematic review. For this reason, the conclusions deduced within this study are limited to a highly variable level of detail and a low level of evidence of the included studies.

## Figures and Tables

**Table 1:** Demographic Data.

Ref.	Year of Publication	Manuscript Category	N° Patients/Procedures	Age (Mean)	mCMS
Tian J et al. [26]	2022	Retrospective	25/25	33	54
Ahn JH et al. [27]	2019	Retrospective	514/514	37.2	48
Quayle J et al. [28]	2018	Retrospective	50/50	57	42
Duan X et al. [29]	2016	Prospective	68/68	59	63
Abdelatif NM et al. [8]	2014	Prospective	19/19	29	62
Bai Z et al. [30]	2013	Retrospective	10/10	27.6	36
Young BH et al. [11]	2011	Retrospective	294/294	37	52
Dannawi Z et al. [31]	2011	Retrospective	55/55	63	55
Kim ES et al. [32]	2011	Retrospective	28/28	-	46
Galla M et al. [33]	2010	Prospective	30/30	49	63
Gougoulias NE et al. [34]	2007	Retrospective	74/78	54	48
Winson IG et al. [35]	2005	Retrospective	116/118	57	50
Pierre A et al. [36]	2003	Prospective	20/20	55	56
Rasmussen S et al. [19]	2002	Retrospective	105/105	35	63
Cameron SE et al. [37]	2000	Retrospective	15/15	51	42
Rolf C et al. [38]	1996	Prospective	109/112	31	65
Crosby LA et al. [39]	1996	Prospective	42/42	46	63
Ogilvie-Harris DJ et al. [40]	1993	Retrospective	17/17	31	53
Thein R et al. [23]	1992	Prospective	9/9	23	45
Barber F A et al. [14]	1990	Prospective	49/53	33	42
Martin D F et al. [3]	1989	Retrospective	57/58	32	58
Total			1706/1720	42	53

mCMS: Modified Coleman Methodology Score.

**Table 2:** Superficial infections.

Ref.	N° Superficial Infections/Total Infections	Medical Therapy			
		Oral ab	Local ab	Local Care	ns ab
Tian J et al. [26]	1/1	X			
Ahn JH et al. [27]	1/1				X
Quayle J et al. [28]	1/1				X
Duan X et al. [29]	1/1			X	X
Abdelatif NM et al. [8]	1/1			X	X
Bai Z et al. [30]	1/1		X		
Young BH et al. [11]	2/2	X	X		
Dannawi Z et al. [31]	3/3	X			
Kim ES et al. [32]	1/1				X
Gougoulias NE et al. [34]	1/1			X	X
Winson IG et al. [35]	3/4				X
Pierre A et al. [36]	2/2			X	
Crosby LA et al. [39]	4/5	X			
Ogilvie-Harris DJ et al. [40]	1/1				X
Thein R et al. [23]	1/1				X
Barber F A et al. [14]	2/3			X	X
Martin D F et al. [3]	2/4	X			

ns: not specified; ab: antibiotics. The “X” indicates the treatment performed.

**Table 3:** Deep infections.

Ref.	N° Deep Infections/Total Infections	Treatment				
		IV AB	Surgical Debridement	Surgical Deb + IV AB	ns	Open Debridement/Arthroscopic Debridement
Galla M et al. [33]	1/1		X			Open
Winson IG et al. [35]	1/4				X	
Rasmussen S et al. [19]	4/4			X		Arthroscopic
Cameron SE et al. [37]	2/2		X			Open
Rolf C et al. [38]	1/1			X		ns
Crosby LA et al. [39]	1/5		X			Open
Barber F A et al. [14]	1/3				X	
Martin D F et al. [3]	2/4			X		ns

IV AB: intravenous antibiotics; ns: not specified. The “X” indicates the treatment performed.

## Data Availability

No new data were created or analyzed in this study. Data sharing is not applicable to this article.
